# Retrospective cohort study evaluating patient-reported outcomes following intensive electromyography and video-biofeedback training in chronic non-flaccid facial palsy

**DOI:** 10.3389/fneur.2026.1759106

**Published:** 2026-02-10

**Authors:** Annika Kunzler, Susanne Hesse, Katharina Geißler, Jonas Ballmaier, Helene Kreysa, Christian Dobel, Susanne Saal, Orlando Guntinas-Lichius, Gerd Fabian Volk

**Affiliations:** 1 Department of Otorhinolaryngology, Jena University Hospital, Jena, Germany; 2 Facial-Nerve-Center, Jena University Hospital, Jena, Germany; 3 Center for Rare Diseases, Jena University Hospital, Jena, Germany; 4 Department for General Psychology and Cognitive Neuroscience, Friedrich Schiller University, Jena, Germany; 5 Department of Health and Nursing, Ernst Abbe University of Applied Sciences Jena, Jena, Germany

**Keywords:** chronic facial palsy, EMG biofeedback, facial paralysis, patient-reported outcomes, quality of life, rehabilitation, synkinesis

## Abstract

**Background:**

Chronic non-flaccid peripheral facial palsy is frequently associated with synkinesis, residual motor deficits, and reduced quality of life. Evidence-based, standardized rehabilitation protocols remain limited. This study examined the effect of an intensive two-week electromyography (EMG) and video-based biofeedback program on changes in physical and psychosocial patient-reported outcomes in patients with chronic facial palsy.

**Methods:**

This retrospective cohort study included patients with chronic non-flaccid facial palsy, either with synkinesis or residual hypotonia, who completed a two-week EMG and video-biofeedback program between 2020 and 2023. The intervention targeted synkinetic co-activation and improved voluntary motor control in hypotonia. Data consisted of routine documentation and patient-reported outcome measures (PROMs) collected at baseline 6 months before therapy (T1), therapy initiation (T2), therapy conclusion (T3), and six-month follow-up (T4). PROMs included the Facial Disability Index (FDI), Facial Clinimetric Evaluation Scale (FaCE), Short Form-36 Health Survey (SF-36), and Beck Depression Inventory (BDI). Analyses used repeated-measures ANOVA and segmented regression.

**Results:**

A total of 175 patients were included. Significant improvements were observed across all PROMs. From T2 to T3, FDI total score increased by 5.24 points (95% CI 3.90 to 6.60) and FaCE total score by 9.86 points (95% CI 7.80 to 11.92). SF-36 showed improvements in social functioning (+4.67 points, 95% CI 2.19 to 7.15) and emotional well-being (+4.32 points, 95% CI 2.60 to 6.00). BDI decreased by 3.29 points (95% CI –4.63 to −1.96). Segmented regression indicated small but significant pre-therapy improvements from T1 to T2. At follow-up, outcomes remained above baseline, with FDI total score rising from 65.64 ± 14.88 at T1 to 75.31 ± 13.83 at T4 and FaCE total score from 53.89 ± 16.54 to 63.72 ± 17.10 (all *p* < 0.001). Older age was associated with lower FDI values; male gender was associated with higher FDI and FaCE and lower BDI scores.

**Conclusion:**

Participation in an intensive EMG- and video-based biofeedback program was associated with clinically relevant and sustained improvements in facial function, quality of life, and psychosocial well-being in patients with chronic non-flaccid facial palsy. Age- and gender-related differences highlight the importance of individualized rehabilitation approaches.

## 1 Introduction

Chronic peripheral facial palsy develops in approximately 30% of cases of acute idiopathic facial palsy (1–5). However, complete absence of regeneration, leading to chronic flaccid paralysis, is very rare. Instead, chronic facial palsy is characterized by incomplete recovery of facial motor function, leading to permanent deficits in the form of aberrant and synkinetic facial reinnervation (6). These changes result in both physical and psychosocial impairments: Patients experience asymmetric appearance, incomplete eye closure, difficulties with eating, drinking, speaking, and lacrimation, as well as decreased social and psychological well-being (7–11).

Many patients with synkinetic aberrant reinnervation present with an increased resting muscle tone, a condition commonly referred to as a hypertonic hemiface. In contrast, a minority of patients exhibit a reduced, but not completely flaccid, resting tone. To distinguish this phenotype from complete flaccidity (as seen in chronic flaccid paralysis), the term “hypotone” is used in this study to describe reduced resting facial muscle tone with preserved voluntary motor activity. This descriptive terminology reflects common clinical usage and is applied consistently throughout the manuscript.

Various treatment options are available for facial palsy, including botulinum toxin injections, physical therapy, mirror therapy, electrical stimulation, mime therapy, surgery, and neuromuscular biofeedback therapy (12). However, the international literature still lacks consensus on optimal rehabilitation protocols regarding indication, intensity, duration, and outcome assessment in chronic facial palsy. Previous work has suggested that rehabilitation may provide functional benefits even in patients with long-standing paralysis (13), but these studies often employed heterogeneous interventions with variable intensity, limiting comparability and generalizability.

In recent years, neuromuscular biofeedback has emerged as a promising rehabilitation approach for motor disorders, including stroke, pain syndromes, and neuromuscular dysfunctions (14–18). This training leverages surface electromyography (EMG) to provide real-time feedback on muscle activity, which is often displayed visually or acoustically (19). This method promotes active patient engagement in retraining motor patterns and improving voluntary muscle control. Despite its proven utility in other neuromuscular conditions, its application to chronic facial palsy remains underexplored.

Since October 2012, the Facial-Nerve-Center Jena at Jena University Hospital, Jena, Germany, has implemented a two-week intensive biofeedback training program for patients with chronic facial palsy. This program addresses both facial synkinesis and hypotonic facial paresis with some remaining motor control, combining neuromuscular retraining with simultaneous EMG and video feedback of facial muscle activities. Unlike classic facial therapy sessions, which typically involve 45–60 min of weekly training, this program is based on the theoretical assumption that increased intensity and repetition may facilitate motor relearning in chronic facial palsy. During the 2 weeks of training, patients participate in guided training for 3 hours per day, supplemented by 2 hours of individual practice per day, thus dedicating approximately 30% of their waking hours to retraining facial movements. After the two-week program, patients are encouraged to continue daily home exercises, including relaxation techniques, for at least 15 min per day.

These principles align with constraint-induced movement therapy (CIMT) and massed practice (20–22). Thus, the program’s design is informed by general motor learning research, which suggests that complex motor patterns are acquired through continuous, intensive practice (22, 23). Similarly, retraining facial movement patterns demands sustained, focused effort, but, to date, data on the application of such training principles to facial motor rehabilitation remains limited.

While other studies have provided valuable insights into facial rehabilitation and the potential benefits of EMG- and video-feedback training (24–26), many of these investigations were constrained by small sample sizes, heterogeneous interventions, or relied primarily on clinician-rated or motor outcomes, without integrating broader patient-reported measures. Building on this important groundwork, the present study extends the evidence base by evaluating a larger cohort of patients with chronic facial palsy (*n* = 175) using a standardized, intensive two-week EMG and video feedback program. In addition, it incorporates a comprehensive set of validated patient-reported outcome measures (PROMs), thereby capturing not only functional but also psychosocial and psychological dimensions of recovery (27–30). The retrospective cohort study, based on routinely collected clinical and PROM data at four distinct time points, allows differentiation between pre-therapy developments, immediate post-intervention gains, and long-term maintenance of reported improvements. We hypothesized that patient-reported facial function and quality of life would improve following the intervention and would remain stable at follow-up.

## 2 Methods

### 2.1 Study design and setting

Our study is based on existing clinical and patient-reported outcome data from the Facial-Nerve-Center of Jena University Hospital, Jena, Germany, covering the period between January 2020 and December 2023. Ethics approval was obtained from the University Ethics Committee of Jena University Hospital (No. 4370–03/15). The primary objective was to evaluate changes in patient-reported outcomes associated with participation in an intensive EMG- and video-based biofeedback training program for chronic peripheral facial palsy. Given the retrospective observational design without a control group, analyses are limited to associations rather than causal effects.

All data were collected at four predefined clinical time points (T1-T4) as part of standardized diagnostic and follow-up procedures. T1 represented routine clinical assessment 6 months prior to the start of the training therapy; T2 was assessed at training initiation; T3 reflected the end of the two-week training intervention; and T4 represented follow-up 6 months after the training program.

### 2.2 Participants

A total of 175 patients with chronic peripheral facial palsy were included in the study. Inclusion criteria were as follows:

- Onset of facial palsy at least 1 year prior to T1.
- Stable disease status, without noticeable improvement in the last 6 months based on the self-assessment of the patients.
- Diagnosis of severe synkinesis (hypertonus) or hypotonic facial palsy.
- Reinnervation of affected facial muscles confirmed via needle EMG.

Patients with incomplete datasets or training therapy durations under 5 days were excluded.

### 2.3 Intervention

The training program consisted of a two-week intensive rehabilitation schedule, spanning 10 consecutive weekdays with a weekend break between the first and second week. Each session lasted for 3 hours and was carried out either individually or in pairs of patients with similar severity of symptoms. Therapy was instructed continuously by one of three specialized therapists (one physiotherapist and two occupational therapists), ensuring consistency and tailored guidance.

**Figure 1:**
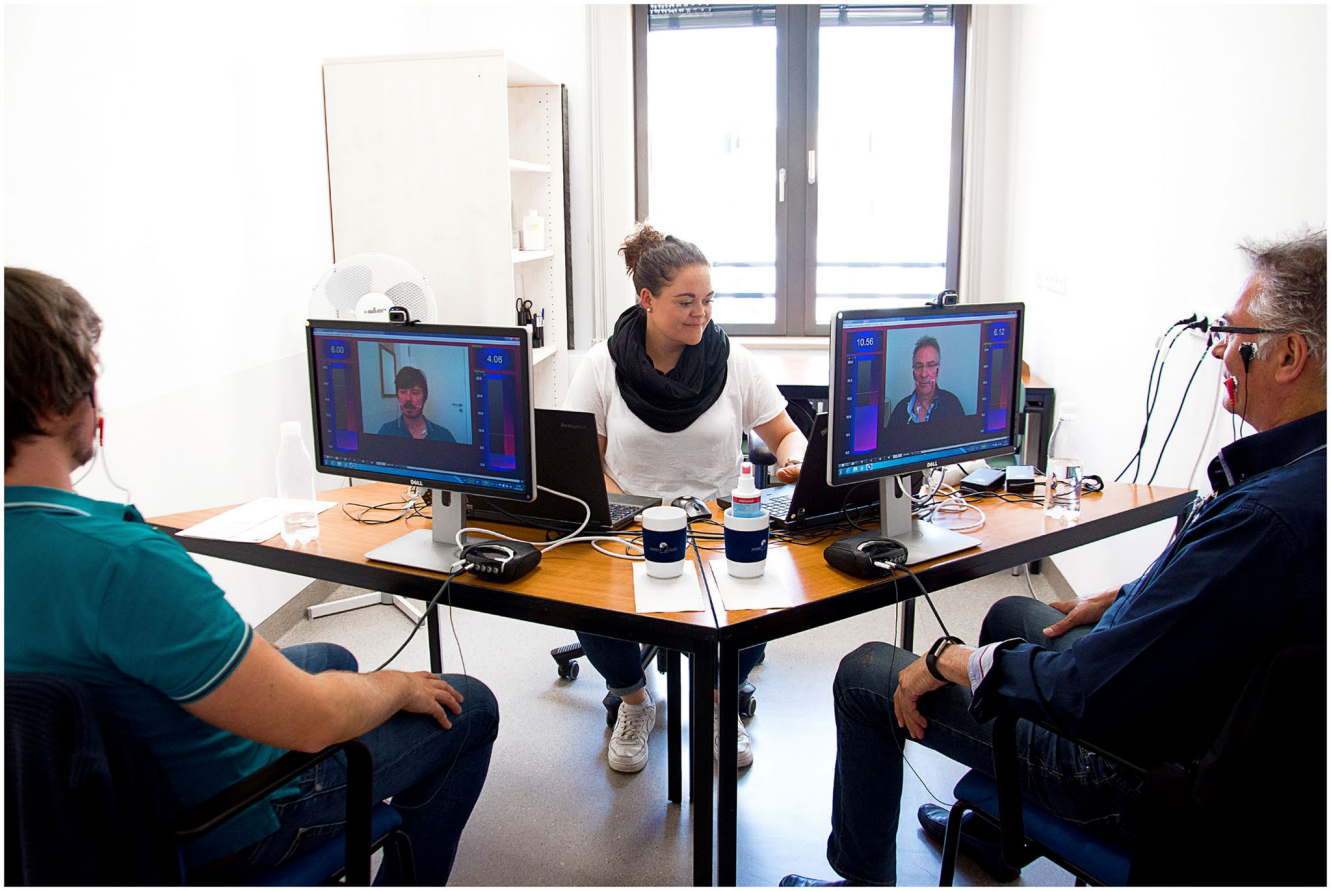
Training setting. The typical training setup involved two patients and therapist seated opposite each other. Each patient was equipped with an individual screen. A webcam-based mirror image of the patient’s face was displayed in the center of the screen, flanked by two vertical electromyographic feedback bars that reflected the activity recorded via adhesive electrodes. Patients saw only their own display, while the therapist had access to both patients’ screens.

The training incorporated EMG and video biofeedback. Electrodes were placed over the orbicularis oris and zygomatic muscles to continuously monitor and record facial muscle activity of the cheek region. Patients were provided with simultaneous video and EMG feedback to observe and refine their facial movements, emphasizing the distinction between intended and unintended muscle activations. This feedback was crucial in normalizing muscle activity, reducing synkinesis and improving voluntary motor control (Figure 1).

In addition to the instructed sessions, patients engaged in 2 hours of self-guided training each afternoon. This training included repetition of the exercises performed earlier that day using mirror feedback to maintain proper technique. Patients were required to document the type and duration of these exercises in a training diary, fostering adherence and enabling monitoring of additional practice.

Following discharge, patients were instructed to continue daily home training for at least 6 months. This aim of this instruction was to maintain the gains achieved during the supervised sessions; it included relaxation exercises for a minimum of 15 min per day, supported by mirror feedback to ensure movement precision. However, the actual extent and consistency of home-based training were not systematically recorded, which limits conclusions about adherence.

The primary goal of the training was to enable patients to achieve functional improvements in facial symmetry and coordination, as well as to reduce synkinesis and overall disability. The structured combination of guided and independent training ensured a comprehensive approach addressing the diverse presentations of facial palsy.

### 2.4 Outcome measures

To evaluate the effects of the therapy, four validated patient-reported outcome measures (PROMs) were used: The Facial Disability Index (FDI), which measures the physical and social impacts of facial paralysis (27); the Facial Clinimetric Evaluation Scale (FaCE), which assesses quality of life across six domains (28); the Short Form-36 Health Survey (SF-36), which evaluates health-related quality of life across eight domains (29), and the Beck Depression Inventory (BDI) which captures the severity of depressive symptoms based on 21 items (30).

These questionnaires were completed at T1, T2, T3, and T4 to capture changes over time. The PROMs were supplemented by clinical observations and detailed reassessments of facial function.

### 2.5 Statistical analysis

Statistical analyses were performed using IBM SPSS Statistics Version 29.0 (IBM Corp., Armonk, NY). Unless otherwise specified, data consisted of mean ± standard deviation (SD). To analyze changes over time, repeated-measures analysis of variance (ANOVA) was used. Mauchly’s test was applied to evaluate sphericity, and Greenhouse–Geisser corrections were implemented when violations were detected.

Post-hoc analyses with Bonferroni adjustments were conducted to identify pairwise differences between the four time points. Significance was set at *p* < 0.05, with *p* < 0.01 indicating highly significant results. Effect sizes (partial η^2^) were calculated to estimate the practical relevance of observed differences. Where applicable, results are reported together with corresponding 95% confidence intervals (CIs) to facilitate interpretation.

Due to the requirements of repeated-measures ANOVA, only patients with complete questionnaire data at all four time points (T1 to T4) could be included in the analysis. Of the 175 patients initially enrolled, complete datasets were available for between 120 and 131 patients, depending on the specific PROM analyzed. Missing data occurred predominantly at follow-up (T4) leading to a reduced sample size for ANOVA models. This complete-case approach could introduce selection bias, as patients with missing follow-up data could differ systematically from those with complete datasets. As no imputation was performed, results from repeated-measures ANOVA should be interpreted with caution regarding generalizability.

In addition to the repeated-measures ANOVA, segmented regression models, based on Generalized Estimating Equations (GEE), were used to analyze both immediate and longitudinal effects of the intervention. GEEs accounted for the repeated-measures structure of the data and provided robust estimates of within-patient change while adjusting for covariates such as age, gender, and baseline values. Because the training therapy began at T2, two continuous time variables were created to model PROM development before and after therapy initiation:

- Pre-therapy PROM trajectory (T1 to T2): This variable represents the linear change in PROM scores in the time leading up to therapy. It quantifies how PROMs evolved during the pre-intervention period and reflects natural regression or anticipation-related effects.
- Post-therapy PROM trajectory (T2 onward): This variable represents the linear change in PROM scores after therapy initiation (T3 and T4). It captures whether outcomes improved, stabilized, or declined after the intervention.
- Immediate therapy response (T2-T3): This variable quantifies the short-term change in PROMs through the EMG biofeedback intervention. It isolates the direct within-therapy effect from pre- and post-therapy trajectories.

The GEE analyses used an identity link with normal distribution, and an exchangeable working correlation structure was selected to account for within-subject dependencies. Regression coefficients (B) and their 95% confidence intervals (CIs) were reported to quantify the magnitude and direction of effects. This modeling strategy allows a clear distinction between pre-therapy trends, within-therapy changes, and post-therapy trajectories, ensuring that longitudinal developments are appropriately attributed to the corresponding study phases.

## 3 Results

### 3.1 Patient characteristics

A total of 180 patients were initially screened for eligibility. Four patients were excluded due to incomplete datasets, and one patient discontinued the program after two therapy days. Thus 175 patients with chronic peripheral facial palsy were available for analysis. The majority of the cohort were female (*n* = 132, 75.4%), while male patients accounted for 24.6% of the sample (*n* = 43). Facial palsy was almost evenly distributed between the right (*n* = 81, 46.3%) and left (*n* = 90, 51.4%) side of the face, with a small subset of patients (*n* = 4, 2.3%) exhibiting bilateral involvement.

Regarding etiology, the most common causes were idiopathic facial palsy (*n* = 68, 38.9%), inflammatory conditions (*n* = 49, 28.0%, among these Varicella-Zoster virus reactivation, also called *Ramsay Hunt Syndrome n* = 39, 22.3%), and benign neoplasms (*n* = 42, 24.0%, including vestibular schwannomas *n* = 29, 16.6%). A smaller proportion of patients had traumatic (*n* = 6, 3.4%), congenital (*n* = 2, 1.1%), or post-COVID-19 vaccine-related (*n* = 4, 2.3%) facial palsy. Among malignant causes, adenoid cystic carcinoma and mucoepidermoid carcinoma were observed in a few cases (*n* = 4, 2.3%).

The mean age of the participants was 49.5 ± 14.0 years, with a median of 52 years (range: 19–81 years). The disease duration before entering the study varied considerably, with a mean of 53.1 ± 78.3 months and a median of 23 months (range: 11–546 months), illustrating the chronic nature of the condition among the study population.

In the acute phase, most patients (*n* = 156, 89.1%) presented with incomplete facial paresis, whereas complete facial paralysis was observed in *n* = 19 (10.9%) based on the medical history and the documentation of the pretreating physicians. In the chronic phase, hypertonic facial palsy (synkinesis-dominated presentation) was predominant (*n* = 167, 95.4%), whereas residual facial weakness was less frequent (*n* = 8, 4.6%).

All patients underwent the two-week biofeedback training program, with a median of 10 days (interquartile range (IQR) 10–10; range 5–10 days). Baseline assessment (T1) took place a median of 6 months (IQR 4–9; range 0–30) before therapy initiation (T2). The end-of-therapy assessment (T3) took place immediately after the two-week training period, and follow-up (T4) occurred a median 6 months (IQR 5–7; range 0–40) after completion of therapy (Table 1).

**Table 1:** Patient characteristics.

Characteristic	*n*	%
Total number of patients	175	100.0
Gender		
Female	132	75.4
Male	43	24.6
Side of palsy		
Right	81	46.3
Left	90	51.4
Bilateral	4	2.3
Etiology		
Idiopathic	68	38.9
Inflammatory	49	28.0
Benign neoplasms	42	24.0
Traumatic	6	3.4
Congenital	2	1.1
Post-COVID-19 Vaccine	4	2.3
Malignant Neoplasms	4	2.3
Acute phase facial palsy		
Incomplete	156	89.1
Complete	19	10.9
Chronic phase presentation		
Hypertonic (Synkinesis)	167	95.4
Hypotonic	8	4.6

Characteristic	Mean ± SD	Median (Range)
Age (years)	49.5 ± 14.0	52 (19–81)
Disease duration (months)	53.1 ± 78.3	23 (11–546)
Therapy duration (days)	9.7 ± 0.9	10 (5–10)

*n*, number of patients; %, percentage; SD, Standard Deviation.

### 3.2 Overall PROM improvements (T1 to T4)

**Figure 2:**
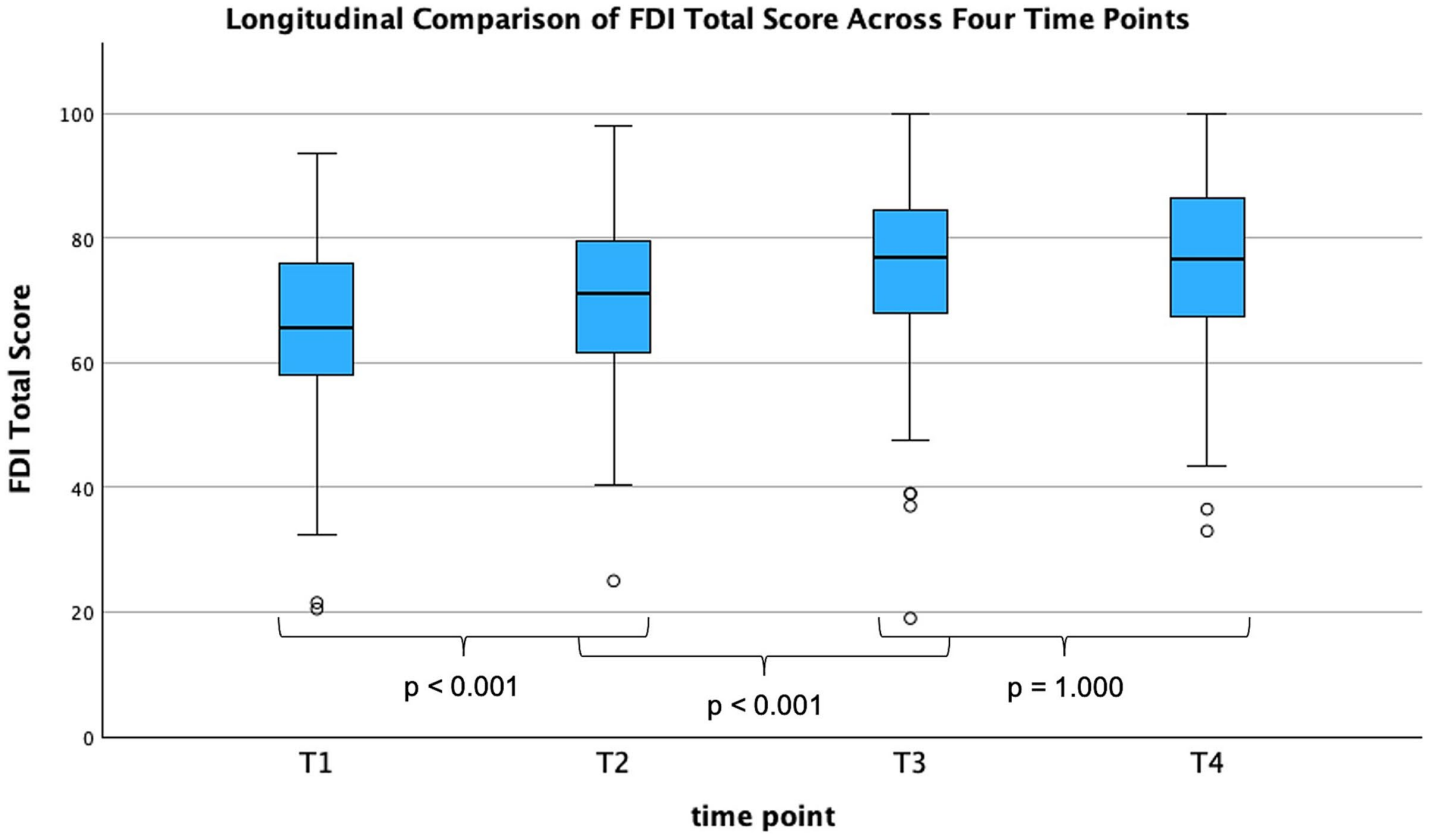
FDI total score across four time points (*n* = 129). Longitudinal development of the Facial Disability Index (FDI) total score across four time points (T1–T4). A repeated-measures ANOVA revealed a significant main effect of time (*F* = 45.61, *p* < 0.001, η^2^ = 0.26). Bonferroni-adjusted pairwise comparisons with showed improvements between T1 and T2 (*p* < 0.001), and between T2 and T3 (*p* < 0.001), while no further change was observed between T3 to T4 (*p* > 0.99). FDI, Facial Disability Index; T, time point; T1, baseline; T2, therapy initiation; T3, therapy conclusion; T4, follow-up; *n*, number of patients; ANOVA, analysis of variance; *F*, F-Statistic; *p*, p-alue; η^2^, partial eta squared.

Across all assessed PROMs, statistically significant changes were observed between the four measurement time points (T1-T4). The Facial Disability Index (FDI) total score, which can reach an ideal maximum of 100, increased from 65.64 ± 14.88 (T1) to 70.29 ± 13.65 (T2), to 75.11 ± 14.08 (T3). By T4, the score remained at a comparable level (75.31 ± 13.83, *n* = 139) (Figure 1). These values correspond to a significant main effect of time in the repeated-measures ANOVA (*F* (2.54, 324.67) = 45.61, *p* < 0.001, η^2^ = 0.26) (Figure 2; Table 2).

**Table 2:** ANOVA results: changes in PROMs across time points.

PROM measure	T1 Mean ± SD	T2 Mean ± SD	T3 Mean ± SD	T4 Mean ± SD	*F*	*p*	η^2^	Sample size (*n*)
FDI physical function	65.94 ± 15.83	70.63 ± 14.67	75.03 ± 14.46	74.61 ± 14.48	31.93	**< 0.001**	0.20	130
FDI social function	65.23 ± 19.18	69.99 ± 17.03	75.23 ± 17.32	76.05 ± 17.32	34.93	**< 0.001**	0.21	130
FDI total score	65.64 ± 14.88	70.29 ± 13.65	75.11 ± 14.08	75.31 ± 13.83	45.61	**< 0.001**	0.26	129
FaCE facial movement	40.91 ± 22.28	42.80 ± 23.15	49.35 ± 19.46	48.35 ± 22.17	14.07	**< 0.001**	0.11	121
FaCE facial comfort	42.33 ± 24.91	43.32 ± 21.27	63.04 ± 22.84	53.31 ± 26.46	46.94	**< 0.001**	0.27	131
FaCE oral function	67.93 ± 25.28	72.09 ± 23.36	77.13 ± 20.62	75.29 ± 23.15	6.27	**< 0.001**	0.05	129
FaCE eye comfort	50.86 ± 31.67	53.24 ± 28.40	59.26 ± 29.36	59.07 ± 30.66	6.30	**< 0.001**	0.05	131
FaCE lacrimal control	63.01 ± 29.57	61.18 ± 30.23	72.76 ± 25.81	68.90 ± 25.48	7.05	**< 0.001**	0.06	123
FaCE social function	63.91 ± 25.56	69.76 ± 24.86	79.13 ± 22.03	77.50 ± 22.73	20.57	**< 0.001**	0.14	130
FaCE total score	53.89 ± 16.54	56.56 ± 13.89	66.86 ± 15.43	63.72 ± 17.10	44.98	**< 0.001**	0.26	131
SF-36 physical functioning	87.13 ± 16.22	86.65 ± 18.08	88.35 ± 18.19	87.90 ± 19.50	0.91	0.418	0.01	130
SF-36 role limitations due to physical health	62.57 ± 39.38	66.99 ± 39.43	72.07 ± 37.60	72.92 ± 36.51	5.24	**0.002**	0.04	128
SF-36 role limitations due to emotional problems	63.59 ± 42.82	70.77 ± 40.56	76.67 ± 36.81	77.44 ± 36.71	7.09	**< 0.001**	0.05	130
SF-36 energy/fatigue	48.43 ± 18.95	51.06 ± 18.70	57.20 ± 18.37	56.94 ± 20.25	19.29	**< 0.001**	0.13	127
SF-36 emotional well-being	63.16 ± 18.77	67.31 ± 16.96	72.06 ± 17.27	69.61 ± 18.40	16.21	**< 0.001**	0.11	127
SF-36 social functioning	72.77 ± 24.58	77.81 ± 24.27	80.43 ± 23.69	81.59 ± 23.29	7.52	**< 0.001**	0.06	129
SF-36 pain	80.29 ± 22.55	77.79 ± 23.54	81.92 ± 21.59	79.17 ± 22.42	1.66	0.181	0.01	130
SF-36 general health	58.52 ± 18.32	59.15 ± 17.69	62.01 ± 17.52	61.69 ± 20.13	3.02	**0.034**	0.02	129
BDI	10.63 ± 7.47	10.61 ± 7.45	7.28 ± 7.05	8.02 ± 7.82	26.41	**< 0.001**	0.18	120

ANOVA, analysis of variance; PROM, Patient-Reported-Outcome Measure; T1, baseline; T2, therapy initiation; T3, therapy conclusion; T4, follow-up; SD, Standard Deviation; *F*, *F*-statistic; *p*, *p*-value; η^2^, partial eta squared (effect size); *n*, sample size; FDI, Facial Disability Index; FaCE, Facial Clinimetric Evaluation Scale; SF-36, Short Form-36; BDI, Beck Depression Inventory. Bold values indicate statistically significant results (*p* < 0.05).

**Figure 3:**
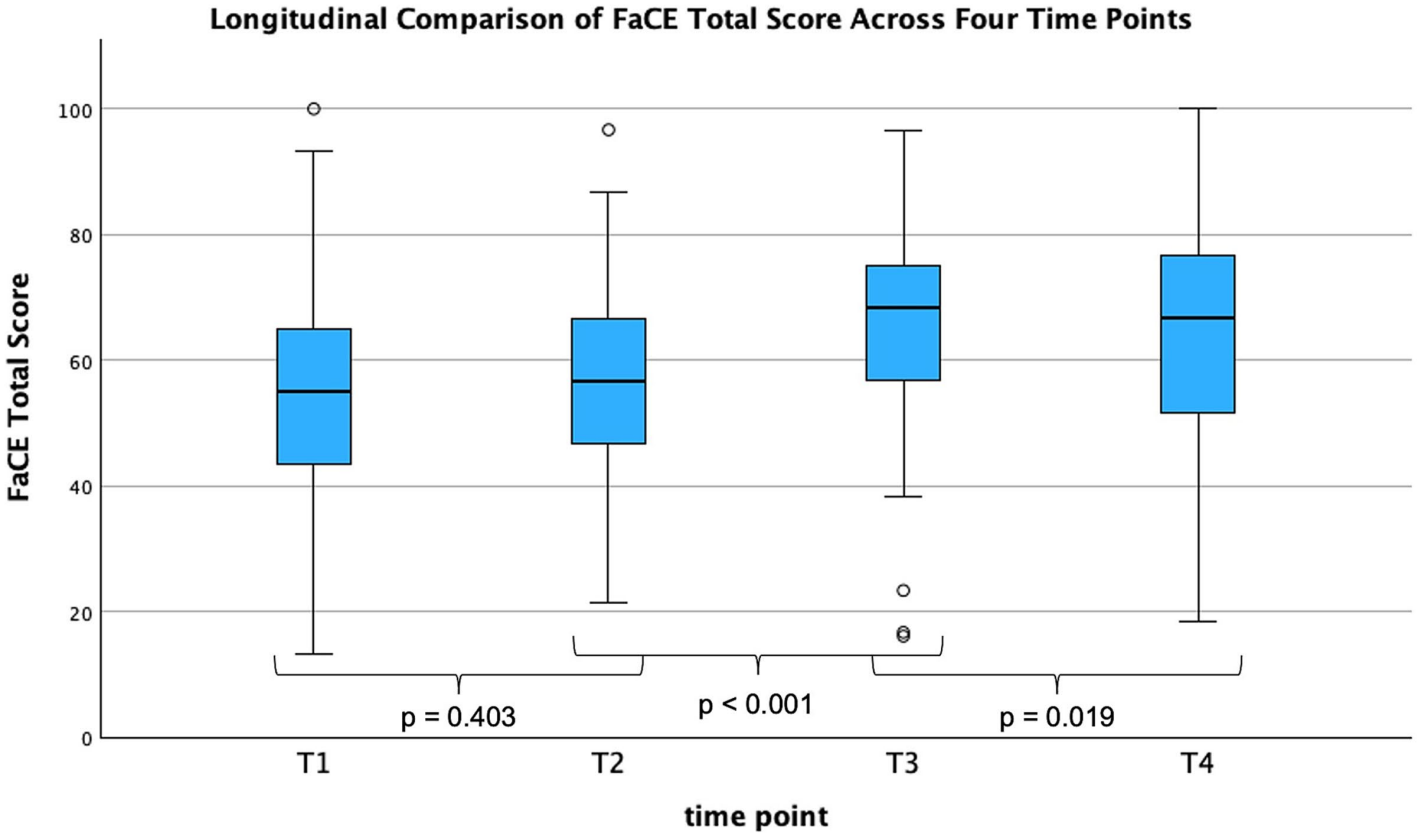
FaCE total score across four time points (*n* = 131). Longitudinal development of the Facial Clinimetric Evaluation Scale (FaCE) total score across four time points (T1–T4). A significant main effect of time was found by repeated-measures ANOVA (*F* = 20.57, *p* < 0.001, η^2^ = 0.14). Bonferroni-adjusted pairwise comparisons revealed a significant increase from T2 to T3 (*p* < 0.001) and from T3 to T4 (*p* = 0.019), while no significant change was observed between T1 and T2 (*p* 0 = 0.403). FaCE, Facial Clinimetric Index; T, time point; T1, baseline; T2, therapy initiation; T3, therapy conclusion; T4, follow-up; n, number of patients; ANOVA, analysis of variance; *F*, *F*-tatistic; *p*, *p*-Value; η^2^, partial eta squared.

The Facial Clinimetric Evaluation Scale (FaCE), which can reach an ideal maximum of 100, increased from 53.89 ± 16.54 at T1 to 56.56 ± 13.89 at T2 to 66.86 ± 15.43 at T3. A lower value was recorded at T4 (63.72 ± 17.10, *n* = 131), yet the T4 score remained higher than baseline. The ANOVA indicated a significant main effect of time (*F* (2.89, 363.77) = 44.98, *p* < 0.001, η^2^ = 0.26) (Figure 3).

Within the FaCE subscales, the facial movement domain increased from 40.91 ± 22.28 (T1) to 42.80 ± 23.15 (T2), 49.35 ± 19.46 (T3), and 48.35 ± 22.17 (T4) (*n* = 121), with a significant effect of time (*F* (3.00, 360.00) = 14.07, *p* < 0.001, η^2^ = 0.11). The social function domain increased from 63.91 ± 25.56 (T1) to 69.76 ± 24.86 (T2), 79.13 ± 22.03 (T3), and 77.50 ± 22.73 (T4) (*n* = 130); the corresponding ANOVA likewise showed a significant effect of time (*F* (2.13, 275.10) = 20.57, *p* < 0.001, η^2^ = 0.14) (Table 2).

The Short Form-36 (SF-36) showed statistically significant changes across several domains. The domain “role limitations due to physical health” increased significantly from 62.57 ± 39.38 (T1) to 72.92 ± 36.51 (T4) (*n* = 128, *F* (2.63, 333.70) = 5.24, *p* = 0.002, η^2^ = 0.04). The “energy/fatigue” domain increased from 51.06 ± 18.70 (T2) to 57.20 ± 18.37 (T3) (*n* = 127, *F* (2.72, 342.77) = 19.29, *p* < 0.001, η^2^ = 0.13) (Table 2).

**Figure 4:**
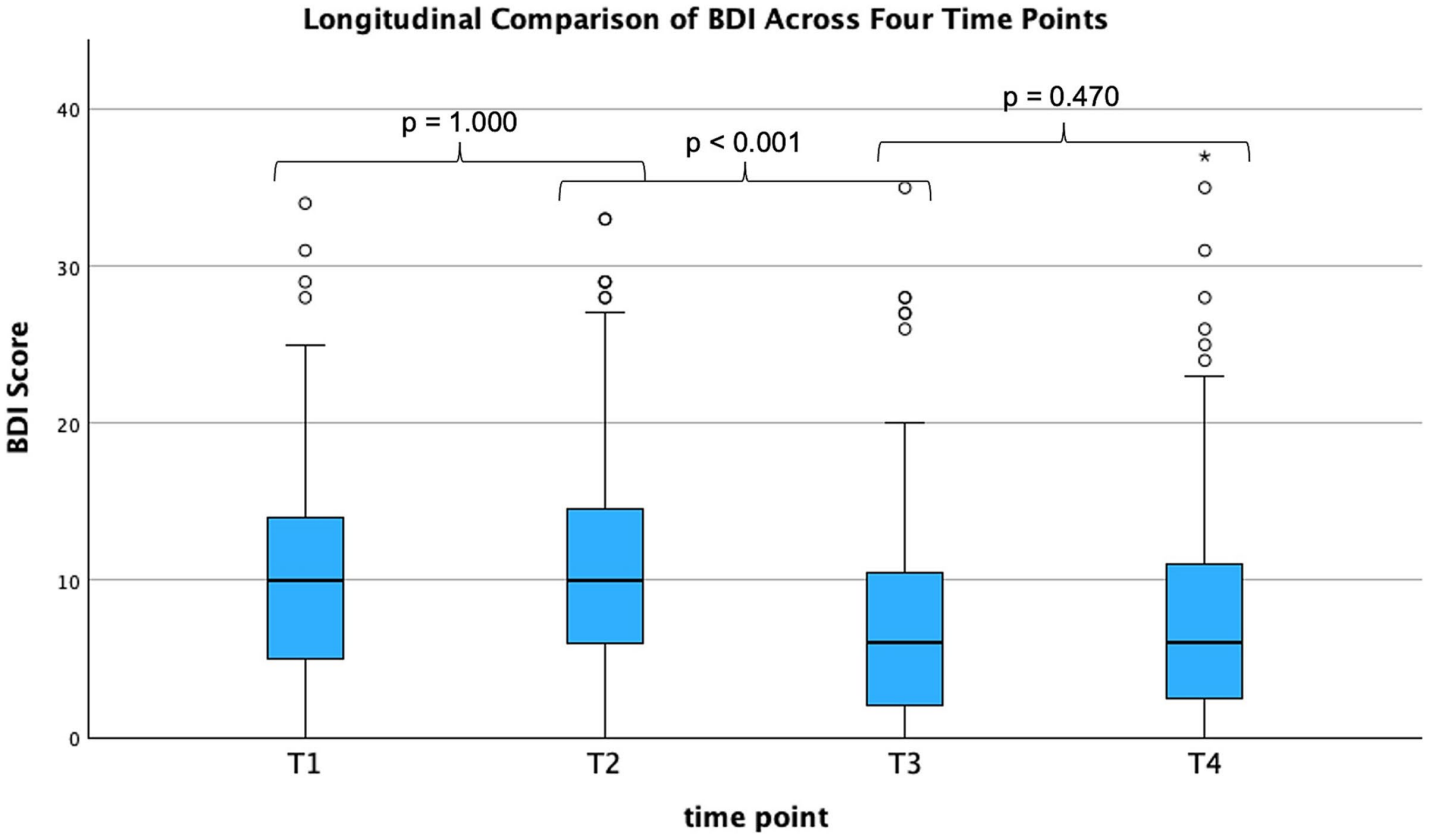
BDI across four time points (*n* = 120). Longitudinal development of Beck Depression Inventory (BDI) scores across four time points (T1–T4). A repeated-measures ANOVA revealed a significant main effect of time (*F* = 26.21, *p* < 0.001, η^2^ = 0.18). Bonferroni-adjusted pairwise comparisons indicated a significant reduction in depressive symptoms between T2 und T3 (*p* < 0.001), with no significant differences observed between T1 and T2 (*p* > 0.99) or T3 and T4 (*p* = 0.470). BDI, Beck Depression Inventory; T, time point; T1, baseline; T2, therapy initiation; T3, therapy conclusion; T4, follow-up; *n*, number of patients; ANOVA, analysis of variance; *F*, F-statistic; *p*, *p*-value η^2^, partial eta squared.

The Beck Depression Inventory (BDI) scores, which can reach a maximum of 63, representing severe depression, also showed numerical changes across time points. Mean values were 10.63 ± 7.47 (T1) and 10.61 ± 7.45 (T2), decreasing to 7.28 ± 7.05 (T3) and 8.02 ± 7.82 (T4) (*n* = 120) (Figure 4). Repeated-measures ANOVA showed a significant main effect of time (*F* (2.84, 337.46) = 26.41, *p* < 0.001, η^2^ = 0.18). Post-hoc tests indicated significant differences between T2 and T3 (*p* < 0.001) (Figure 4; Table 2).

Additional visualizations of overall PROM trajectories, including individual score distributions, are provided in the Supplementary material.

### 3.3 Immediate therapy response (T2 to T3)

Segmental regression analysis showed statistically significant changes in all assessed PROMs from T2 to T3. The FDI total score increased by 5.24 points (95% CI: 3.89 to 6.60, *p* < 0.001). The FaCE total score increased by 9.86 points (95% CI: 7.80 to 11.92, *p* < 0.001). For the SF-36, the social functioning domain increased by 4.67 points (95% CI, 2.19 to 7.15, *p* < 0.001), and the emotional well-being domain increased by 4.32 points (95% CI, 2.60 to 6.03, *p* < 0.001). The BDI decreased by −3.20 points (95% CI, −3.91 to −2.49, *p* < 0.001). These values represent the model-estimated changes from T2 to T3 as quantified by the segmented regression analyses (Table 3).

**Table 3:** Segmented regression analysis: immediate therapy response and longitudinal trends in PROM scores (adjusted for age and gender).

PROM measure	Immediate therapy response (T2 to T3)			Pre-therapy PROM trajectory (T1 to T2)			Post-therapy PROM trajectory (T2 onward)		
	Mean change	95% CI	*p*	Coefficient (Points/Day)	95% CI	*p*	Coefficient (Points/Day)	95% CI	*p*
FDI physical function	+4.08	2.51–5.66	**< 0.001**	+0.020	0.014–0.027	**< 0.001**	−0.016	−0.026–(−0.006)	**0.001**
FDI social function	+6.28	4.60–7.95	**< 0.001**	+0.019	0.011–0.026	**< 0.001**	−0.019	−0.029–(−0.008)	**< 0.001**
FDI total score	+5.24	3.89–6.60	**< 0.001**	+0.019	0.014–0.025	**< 0.001**	−0.018	−0.027–(−0.010)	**< 0.001**
FaCE facial movement	+5.86	3.06–8.66	**< 0.001**	+0.010	0.000–0.020	**0.039**	−0.011	−0.026–0.004	0.154
FaCE facial comfort	+16.23	12.88–19.58	**< 0.001**	+0.005	−0.005–0.016	0.319	−0.030	−0.051–(−0.009)	**0.005**
FaCE oral function	+5.06	1.55–8.57	**0.005**	+0.012	−0.003–0.028	0.125	−0.010	−0.029–0.008	0.279
FaCE eye comfort	+7.98	4.71–11.26	**< 0.001**	+0.004	−0.009–0.017	0.540	−0.007	−0.029–0.015	0.506
FaCE lacrimal control	+9.20	5.29–13.11	**< 0.001**	+0.008	−0.008–0.023	0.332	−0.022	−0.045–0.001	0.063
FaCE social function	+12.15	8.46–15.84	**< 0.001**	+0.006	−0.013–0.024	0.548	−0.008	−0.032–0.015	0.501
FaCE total score	+9.86	7.80–11.92	**< 0.001**	+0.009	0.000–0.018	**0.047**	−0.018	−0.031–(−0.005)	**0.007**
SF-36 physical functioning	+1.90	0.74–3.06	**0.001**	+0.002	−0.005–0.008	0.643	+0.003	−0.009–0.014	0.640
SF-36 role limitations due to physical health	+5.70	2.35–9.06	**< 0.001**	+0.011	−0.007–0.028	0.233	−0.006	−0.033–0.022	0.680
SF-36 role limitations due to emotional problems	+5.02	0.55–9.49	**0.028**	+0.019	−0.002–0.040	0.070	−0.012	−0.040–0.015	0.383
SF-36 energy/fatigue	+5.71	3.87–7.55	**< 0.001**	+0.006	−0.001–0.013	0.087	+0.001	−0.012–0.013	0.919
SF-36 emotional well-being	+4.32	2.60–6.03	**< 0.001**	+0.009	0.002–0.016	**0.011**	−0.011	−0.022–0.001	0.073
SF-36 social functioning	+4.67	2.19–7.15	**< 0.001**	+0.009	−0.003–0.022	0.153	−0.010	−0.028–0.007	0.248
SF-36 pain	+4.76	2.42–7.10	**< 0.001**	−0.012	−0.024–(−0.001)	**0.037**	+0.005	−0.013–0.022	0.597
SF-36 general health	+2.62	1.00–4.24	**0.001**	+0.002	−0.007–0.011	0.668	−0.001	−0.015–0.012	0.841
BDI	−3.20	−3.91–(−2.49)	**< 0.001**	+0.0003	−0.004–0.003	0.876	+0.003	−0.002–0.009	0.263

PROM, Patient-Reported-Outcome Measure; T1, baseline; T2, therapy initiation; T3, therapy conclusion; T4, follow-up; CI, confidence interval; *p*, *p*-value; FDI, Facial Disability Index; FaCE, Facial Clinimetric Evaluation Scale; SF-36, Short Form-36; BDI, Beck Depression Inventory. Bold values indicate statistically significant results (*p* < 0.05).

### 3.4 Longitudinal therapy response (T1 to T2 and T2 onward)

The segmented regression showed statistically significant changes in PROM trajectories before and after therapy. Prior to therapy (pre-therapy PROM trajectory), the FDI total score increased by 0.019 points per day (95% CI: 0.014 to 0.025, *p* < 0.001). The FaCE total score increased by 0.009 points per day (95% CI: 0.000 to 0.018, *p* = 0.047). BDI scores did not show a statistically significant change prior to therapy (*p* = 0.876).

Following therapy (post-therapy PROM trajectory), the FDI total score changed by −0.018 points per day (95% CI: −0.027 to −0.010, p < 0.001), and FaCE total score changed by −0.018 points per day (95% CI: −0.031 to −0.005, *p* = 0.007). BDI scores did not show a statistically significant change during this period (*p* = 0.263) (Table 3).

### 3.5 Subgroup analysis

Subgroup analyses descriptively examined differences in PROM values across demographic and clinical factors.

For age, the FDI total score showed a negative association, decreasing by 0.198 points per year (95% CI: −0.34 to −0.06, *p* = 0.006), indicating that higher age was associated with lower FDI total scores. No statistically significant associations were observed for age in the FaCE total score (*p* = 0.054) or the BDI (*p* = 0.351).

For gender, differences in PROM values were observed across time points. In the FDI total score, males scored 5.11 points higher on average than females (95% CI: 0.91 to 9.31, *p* = 0.017). In the FaCE total score, males scored 5.89 points higher on average compared to females (95% CI: 2.20 to 9.57, *p* = 0.002). BDI scores were lower in males, with a mean difference of −3.20 points (95% CI: −5.38 to −1.02, *p* = 0.004) (Table 4).

**Table 4:** Segmented regression analysis: subgroup analysis by age and gender.

PROM measure	Age			Gender		
	Coefficient (Points/Year)	95% CI	*p*	Difference (M-F)	95% CI	*p*
FDI physical function	−0.336	−0.48–(−0.19)	**< 0.001**	+4.08	−0.42–8.59	0.075
FDI social function	−0.060	−0.23–0.11	0.483	+6.38	1.34–11.43	**0.013**
FDI total score	−0.198	−0.34–(−0.06)	**0.006**	+5.11	0.91–9.31	**0.017**
FaCE facial movement	−0.166	−0.35–0.02	0.074	+4.57	−1.40–10.55	0.133
FaCE facial comfort	+0.046	−0.15–0.24	0.646	+10.46	4.39–16.53	**< 0.001**
FaCE oral function	−0.367	−0.55–(−0.18)	**< 0.001**	+2.39	−2.90–7.68	0.376
FaCE eye comfort	−0.452	−0.72–(−0.19)	**< 0.001**	+8.13	0.27–15.98	**0.043**
FaCE lacrimal control	−0.492	−0.71–(−0.28)	**< 0.001**	−1.77	−8.21–4.68	0.591
FaCE social function	+0.065	−0.11–0.24	0.466	+6.85	1.61–12.09	**0.010**
FaCE total score	−0.123	−0.25–0.00	0.054	+5.89	2.20–9.57	**0.002**
SF-36 physical functioning	−0.392	−0.53–(−0.26)	**< 0.001**	+4.61	0.29–8.94	**0.037**
SF-36 role limitations due to physical health	−0.722	−1.01–(−0.44)	**< 0.001**	+6.25	−3.46–15.96	0.207
SF-36 role limitations due to emotional problems	−0.464	−0.74–(−0.18)	**0.001**	+7.59	−2.04–17.22	0.122
SF-36 energy/fatigue	−0.056	−0.23–0.11	0.514	+7.01	1.33–12.68	**0.016**
SF-36 emotional well-being	−0.074	−0.23–0.09	0.362	+5.68	0.61–10.75	**0.028**
SF-36 social functioning	−0.016	−0.21–0.18	0.872	+6.27	−0.08–12.61	0.053
SF-36 pain	−0.128	−0.32–0.07	0.202	+6.32	0.53–12.10	**0.032**
SF-36 general health	−0.241	−0.41–(−0.07)	**0.005**	+3.34	−2.03–8.71	0.223
BDI	+0.033	−0.04–0.10	0.351	−3.20	−5.38–(−1.02)	**0.004**

PROM, Patient-Reported-Outcome Measure; CI, confidence interval; *p*, *p*-value; M, male; F, female; FDI, Facial Disability Index; FaCE, Facial Clinimetric Evaluation Scale; SF-36, Short Form-36; BDI, Beck Depression Inventory. Bold values indicate statistically significant results (*p* < 0.05).

Disease duration prior to therapy (split half: < 23 months vs. ≥ 23 months) showed no statistically significant group differences for either the FDI total score (*p* = 0.484) or the FaCE total score (*p* = 0.268).

Across the three most common etiologies in this cohort (idiopathic, inflammatory, benign neoplasms), no statistically significant differences were observed in the FDI total score, FaCE total score, any SF-36 subscale, or the BDI.

In subgroup analyses based on facial motor phenotype, no significant group-by-time interactions were found for any PROMs, except for the FaCE subscale ‘Facial Comfort’. In this subscale, patients with synkinetic facial palsy showed significant changes across multiple time points, whereas no significant changes were observed in the hypotonic group. However, due to the small number of hypotonic cases in the repeated-measures analysis (*n* = 5–6, vs. *n* = 115–126 for synkinetic facial palsy), these results should be interpreted with caution (Figure 4).

### 3.6 Sustained effects at T4

Despite the slight decline observed in the post-therapy PROM trajectory, PROM scores at T4 remained significantly different from baseline in a favorable direction. Comparing post-therapy effects, the FDI total score showed no significant change between T3 (75.11 ± 14.08) and T4 (75.31 ± 13.83) (*n* = 129, 95% CI: −2.46 to 2.07, *p* > 0.99). In contrast, the FaCE total score decreased from T3 (66.86 ± 15.43) to T4 (63.72 ± 17.10) (*n* = 131, 95% CI: 0.35 to 5.94, *p* = 0.019), while still substantially exceeding the values of T1 (53.89 ± 14.54) (Table 5). SF-36 scores at T4 remained above baseline levels across several domains, including role limitations due to physical health (*n* = 128, 95% CI: −9.04 to 7.36, *p* > 0.99) and emotional problems (*n* = 130, 95% CI: −9.50 to 7.97, *p* > 0.99), energy/fatigue (*n* = 127, 95% CI: −3.90 to 4.58, *p* > 0.99), emotional well-being (*n* = 127, 95% CI: −1.31 to 6.44, *p* = 0.473), and social functioning (*n* = 129, 95% CI: −6.81 to 4.89, *p* > 0.99). BDI scores showed no significant change between T3 and T4 (*n* = 120, 95% CI: −1.84 to 0.38, *p* = 0.470).

**Table 5:** Pairwise comparisons of FDI total score and FaCE total score across time points (Bonferroni-adjusted for multiple comparisons).

Time point (I)	Time point (J)	FDI total score				FaCE total score				BDI			
		Mean difference (I-J)	Standard error	*p*	95% CI	Mean Difference (I-J)	Standard Error	*p*	95% CI	Mean difference (I-J)	Standard error	*p*	95% CI
T1	T2	−4.65	0.921	**< 0.001**	−7.12–(−2.18)	−2.67	1.447	0.403	−6.55–1.21	0.02	0.495	> 0.999	−1.31–1.34
T1	T3	−9.48	1.116	**< 0.001**	−12.47–(−6.49)	−12.97	1.213	**< 0.001**	−16.21–(−9.72)	3.34	0.500	**< 0.001**	2.00–4.69
T1	T4	−9.67	1.156	**< 0.001**	−12.77–(−6.58)	−9.82	1.160	**< 0.001**	−12.93–(−6.71)	2.61	0.549	**< 0.001**	1.14–4.08
T2	T1	4.65	0.921	**< 0.001**	2.18–7.12	2.67	1.447	0.403	−1.21–6.55	−0.02	0.495	> 0.999	−1.34–1.31
T2	T3	−4.82	0.747	**< 0.001**	−6.83–(−2.82)	−10.29	1.337	**< 0.001**	−13.88–(−6.71)	3.33	0.420	**< 0.001**	2.20–4.45
T2	T4	−5.02	0.925	**< 0.001**	−7.50–(−2.54)	−7.15	1.401	**< 0.001**	−10.90–(−3.40)	2.59	0.481	**< 0.001**	1.30–3.88
T3	T1	9.48	1.116	**< 0.001**	6.49–12.47	12.97	1.213	**< 0.001**	9.72–16.21	−3.34	0.500	**< 0.001**	−4.68–(−2.00)
T3	T2	4.82	0.747	**< 0.001**	2.82–6.83	10.29	1.337	**< 0.001**	6.71–13.88	−3.33	0.420	**< 0.001**	−4.45–(−2.20)
T3	T4	−0.20	0.845	> 0.999	−2.46–2.07	3.14	1.045	**0.019**	0.35–5.94	−0.73	0.413	0.470	−1.84–0.38
T4	T1	9.67	1.156	**< 0.001**	6.58–12.77	9.82	1.160	**< 0.001**	6.71–12.93	−2.61	0.549	**< 0.001**	−4.08–(−1.14)
T4	T2	5.02	0.925	**< 0.001**	2.54–7.50	7.15	1.401	**< 0.001**	3.40–10.90	−2.59	0.481	**< 0.001**	−3.88–(−1.30)
T4	T3	0.20	0.845	> 0.999	−2.07–2.46	−3.14	1.045	**0.019**	−5.94–(−0.35)	0.73	0.413	0.470	−0.38–1.84

T, time point; T1, baseline; T2, therapy initiation; T3, therapy conclusion; T4, follow-up; CI, confidence interval; *p*, *p*-value; FDI, Facial Disability Index; FaCE, Facial Clinimetric Evaluation Scale; BDI, Beck Depression Inventory. Bold values indicate statistically significant results (*p* < 0.05).

## 4 Discussion

This study retrospectively examined changes in patient-reported outcomes in 175 individuals with chronic peripheral facial palsy who completed a standardized two-week EMG- and video-based biofeedback program. Using validated instruments (FDI, FaCE, SF-36, BDI), statistically significant changes were observed across functional, psychosocial, and psychological domains. A small but statistically significant improvement occurred during the pre-therapy period (T1–T2). Given the chronic stage of disease, this change is unlikely to reflect spontaneous neurological recovery and may instead be explained by non-specific influences such as expectancy effects, regression to the mean, or low-intensity outpatient therapy recommended at initial presentation. Notably, the magnitude of these changes was modest compared with the substantially larger improvements observed during the intensive two-week biofeedback program.

Additional contextual factors may have contributed to the observed improvements, including increased therapist-patient interaction during the intensive training phase, heightened self-awareness of facial movements through continuous visual and EMG feedback, and psychosocial adaptation associated with structured engagement in a specialized rehabilitation setting. While these influences cannot be disentangled from intervention-specific effects in the present design, acknowledging their potential contribution is important when interpreting the observed associations.

Improvements were evident from baseline to the end of the therapy, and although the scores partially declined thereafter, they remained above baseline at follow-up. Subgroup analyses showed that higher age was associated with lower overall PROM scores, while male patients showed higher FDI and FaCE values and lower BDI scores across time points.

Previous studies have demonstrated that biofeedback-based rehabilitation can improve facial motor function and synkinesis in patients with chronic facial palsy, although most were limited by small sample sizes, heterogeneous protocols, or a narrow focus on clinician-rated outcomes (13, 26, 31). More recent work has expanded this field by incorporating intensified EMG- and video-based training as well as objective assessment tools, demonstrating improvements in facial grading and providing quantitative insights into aberrant motor control in synkinesis (24, 25, 32). In contrast to much of the existing literature, the present study applied a standardized, high-intensity EMG- and video-based protocol in a large real-world cohort and combined objective clinical assessment with a comprehensive PROM battery. This approach allows for a broader characterization of the functional, psychosocial, and psychological outcome trajectories associated with intensive biofeedback training.

A key feature of the present study is the massed-practice structure of the intervention, combining several hours of guided training with additional self-practice. This high-intensity format aligns with established neurorehabilitation principles that emphasize repetition and task-specific training as potential contributors to motor relearning (20, 33, 34). Continuous visual and EMG-based feedback may further enhance patients’ perceived control over facial movements, potentially strengthening self-efficacy, which has been linked to engagement, persistence, and patient-reported outcomes in neurorehabilitation (35, 36).

The large cohort enabled detailed modeling of PROM trajectories, as well as subgroup analyses. Associations between age, gender, and PROM scores were observed and should be interpreted descriptively, as the study was not designed to identify potential underlying mechanisms. These differences may reflect psychosocial factors, baseline symptom burden, coping strategies, or unmeasured confounders rather than biological effects of the intervention. Consistent with mixed evidence in the literature, older patients showed lower overall PROM scores, while male patients showed higher FDI and FaCE scores and lower BDI scores, underscoring the need for further prospective studies to clarify demographic influences (37, 38).

The systematic use of PROMs further strengthens this work. By capturing functional, psychosocial, and emotional dimensions of recovery, PROMs complement clinician-rated assessments and allow for a more comprehensive evaluation of patient-reported change (28).

Improvements in psychosocial domains were reflected in both SF-36 and BDI outcomes. Chronic facial palsy is frequently associated with social withdrawal, reduced quality of life, and mood disturbances (8, 39, 40). The observed gains in energy/fatigue, emotional well-being, and depressive symptoms are consistent with improvements beyond motor domains and suggest that intensive biofeedback training may address broader psychosocial dimensions relevant to patient experience (41, 42).

Despite these strengths, several limitations must be acknowledged. The retrospective design precludes causal inference, and because EMG and video feedback were delivered simultaneously, the contribution of each cannot be isolated. Given that chronic facial palsy is a rare condition and all included patients had already reached the chronic stage months to years after the initial nerve injury, the retrospective cohort approach was considered appropriate for examining within-patient changes. Nonetheless, the inherent constraints of non-experimental designs remain, and randomized or prospective studies are required to confirm the differential contributions of individual therapy components and long-term effects.

While the inclusion of a non-treatment control group could provide further insight into intervention-specific effects, such a design raises ethical concerns in this patient population, given the chronic nature of facial palsy and its associated psychosocial burden.

The slight decline observed in PROM scores after therapy highlights the challenges of sustaining improvements over time and is consistent with previous observations that the absence of guidance may lead to regression in motor control (14). Periodic follow-ups, booster sessions, or digital tools may help support long-term engagement. Although adherence to home-based training was not systematically assessed, difficulties in maintaining unsupervised practice may partly explain the observed post-therapy decline.

Taken together, the present findings should be interpreted as associations between participation in an intensive EMG- and video-based biofeedback program and improvements in patient-reported outcomes. While causal conclusions cannot be drawn, the magnitude and consistency of changes observed across multiple PROMs, particularly during the intervention phase, support further investigation in prospective controlled studies.

## 5 Conclusion

This retrospective cohort study found associations between participation in an intensive two-week EMG- and video-based biofeedback program and improvements in physical, psychosocial, and psychological patient-reported outcomes in patients with chronic peripheral facial palsy. These findings are consistent with the initial hypothesis that structured, high-intensity biofeedback training may be associated with meaningful improvements even in long-standing disease. Although PROM scores declined modestly after therapy, the outcome measures remained above baseline at follow-up after approximately half a year, suggesting persistence of reported benefits.

## Data Availability

The datasets presented in this article cannot be made publicly available due to patient confidentiality and institutional data protection policies. Requests to access the datasets should be directed to the corresponding author.
