# Supplementary material for: Retrospective cohort study evaluating patient-reported outcomes following intensive electromyography and video-biofeedback training in chronic non-flaccid facial palsy
Source: Front Neurol. 2026 Feb 10;17:1759106. doi: 10.3389/fneur.2026.1759106 (PMC12929104; doi:10.3389/fneur.2026.1759106)
Supplement: Supplementary file 1 [file Data_Sheet_1.DOCX]

Supplementary Material

## Supplementary Figures


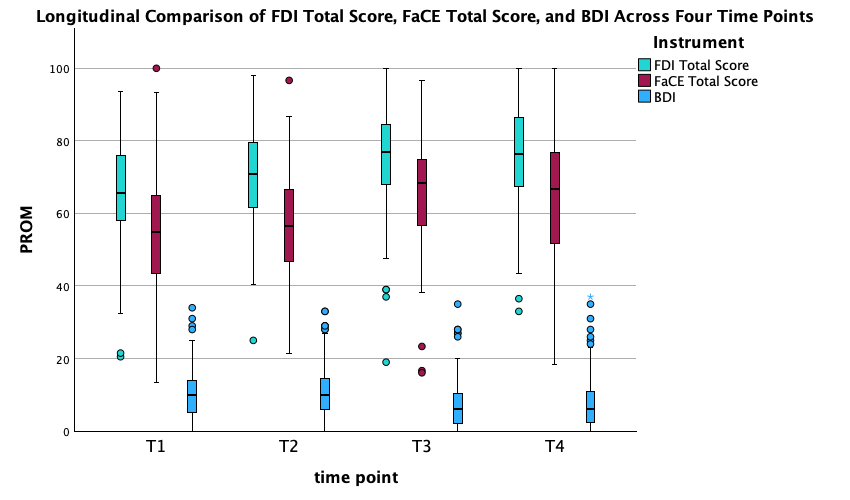


Supplementary Figure 1: FDI Total Score (n = 129), FaCe Total Score (n = 131), and BDI (n = 120) across four time points

Longitudinal comparison of FDI Total Score, FaCE Total Score, and BDI across four time points (T1–T4). All three PROMs showed significant main effects of time in repeated measures ANOVA (FDI: F = 45.61, p < 0.001, η² = 0.26; FaCE: F = 20.57, p < 0.001, η² = 0.14; BDI: F = 26.41, p < 0.001, η² = 0.18). Post-hoc comparisons (Bonferroni-adjusted) revealed significant improvements between consecutive time points for FDI and FaCE, and a significant reduction in depressive symptoms (BDI) between T2 and T3.

Abbreviations: FDI = Facial Disability Index; FaCE = Facial Clinimetric Index; BDI = Beck Depression Inventory; PROM = Patient-Reported-Outcome Measure; T = time point; T1 = baseline; T2 = therapy initiation; T3 = therapy conclusion; T4 = follow-up; n = number of patients; ANOVA = analysis of variance; F = F-Statistic; p = p-Value; η^2^ = partial eta squared


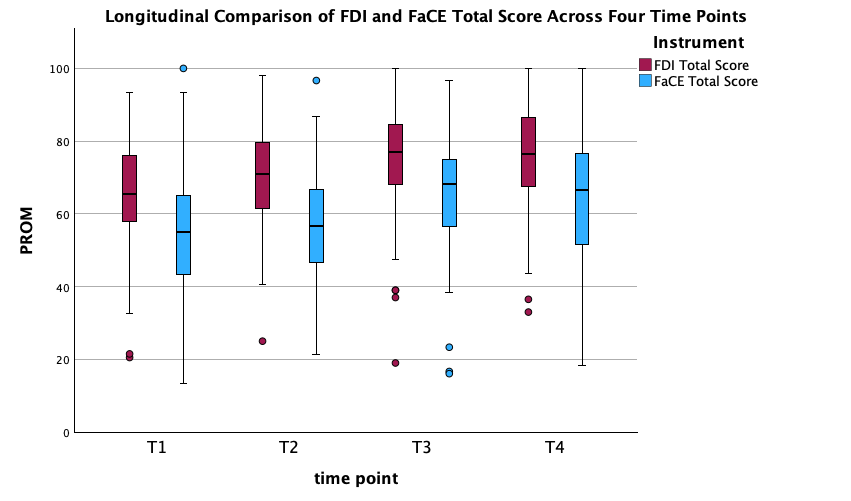


Supplementary Figure 2: FDI Total Score (n =129) and FaCE Total Score (n = 131) across four time points

Longitudinal comparison of FDI Total Score and FaCE Total Score across four time points (T1–T4). Both PROMs showed a significant main effect of time in repeated measures ANOVA (FDI: F = 45.61, p < 0.001, η² = 0.26; FaCE: F = 20.57, p < 0.001). Bonferroni-adjusted post-hoc comparisons indicated significant improvements between consecutive time points for both measures.

Abbreviations: FDI = Facial Disability Index; FaCE = Facial Clinimetric Index; PROM = Patient-Reported-Outcome Measure; T = time point; T1 = baseline; T2 = therapy initiation; T3 = therapy conclusion; T4 = follow-up; n = number of patients; ANOVA = analysis of variance; F = F-Statistic; p = p-Value; η^2^ = partial eta squared


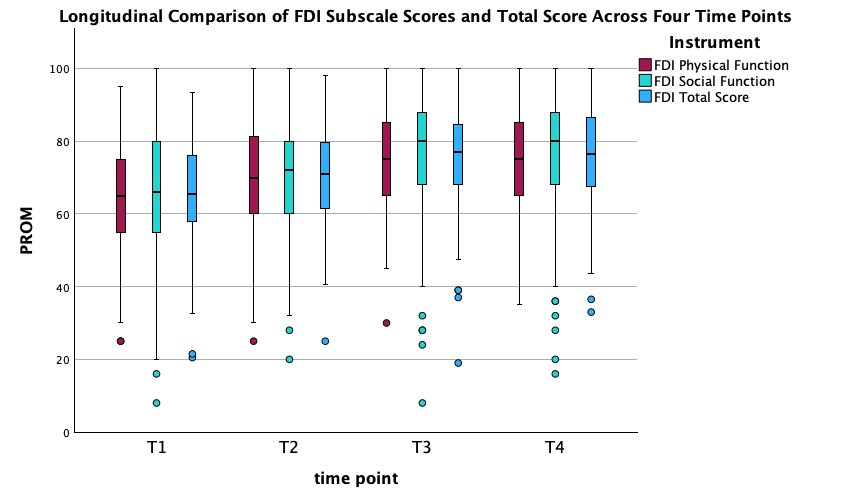


Supplementary Figure 3: FDI Physical Function (n = 130) and Social Function (n = 130) subscale and FDI Total Score (n = 129) across four time points

Longitudinal comparison of the FDI Physical Function and Social Function subscales as well as the FDI Total Score across four time points (T1–T4). All three measures showed a significant main effect of time in repeated measures ANOVA (all p < 0.05). Bonferroni-adjusted post-hoc comparisons revealed significant improvements between T2 and T3 (therapy) for both subscales and the Total Score. No significant changes were observed between T3 and T4, suggesting a stabilization and maintenance of treatment effects following the intervention.

Abbreviations: FDI = Facial Disability Index; PROM = Patient-Reported-Outcome Measure; T = time point; T1 = baseline; T2 = therapy initiation; T3 = therapy conclusion; T4 = follow-up; n = number of patients; ANOVA = analysis of variance; p = p-Value


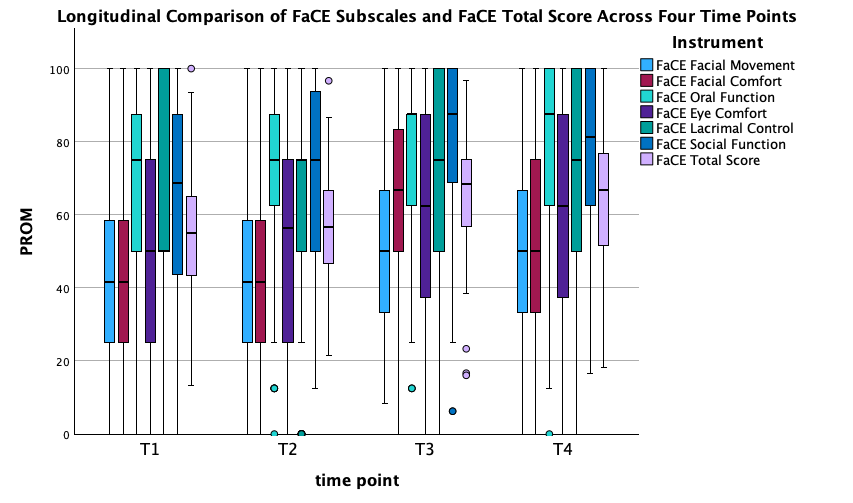


Supplementary Figure 4: FaCE subscales (n = 121 – 131) and FaCE Total Score (n = 131) across four time points

Longitudinal comparison of FaCE subscale scores and the FaCE Total Score across four time points (T1–T4). All subscales and the Total Score showed a significant main effect of time in repeated measures ANOVA (p < 0.05). Bonferroni-adjusted post-hoc comparisons between T2 and T3 (therapy) revealed significant improvements in all subscales except for Oral Function and Eye Comfort, which did not reach statistical significance.

Abbreviations: FaCE = Facial Clinimetric Index; PROM = Patient-Reported-Outcome Measure; T = time point; T1 = baseline; T2 = therapy initiation; T3 = therapy conclusion; T4 = follow-up; n = number of patients; ANOVA = analysis of variance; p = p-Value

Supplementary Figure 5: FaCE subscales and FaCE Total Score across four time points


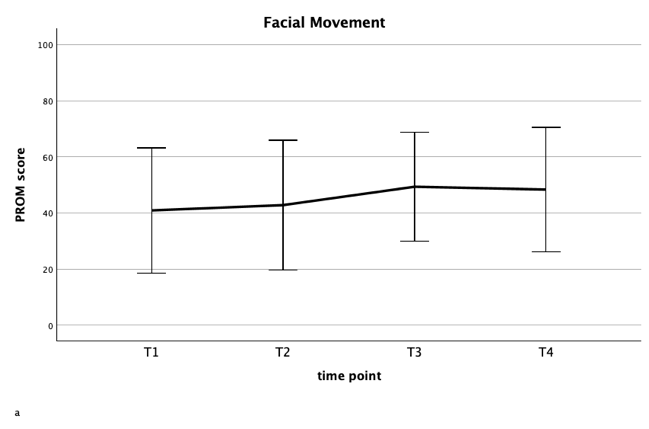

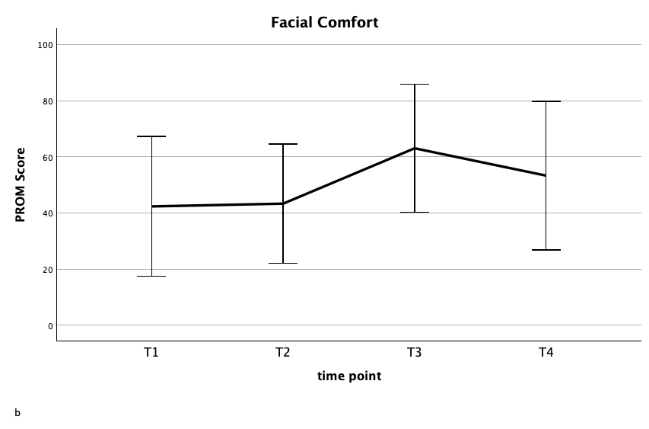

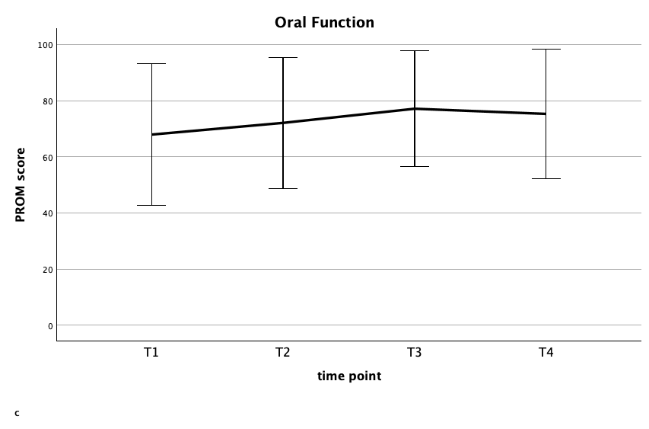

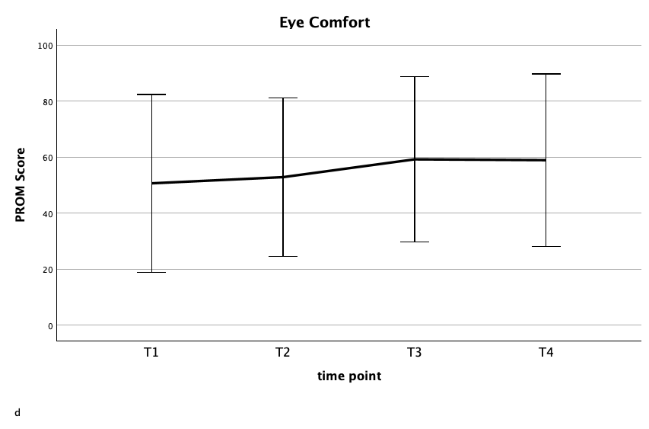

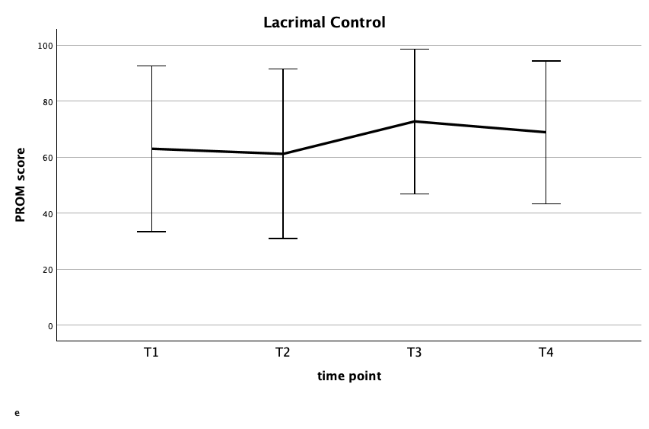

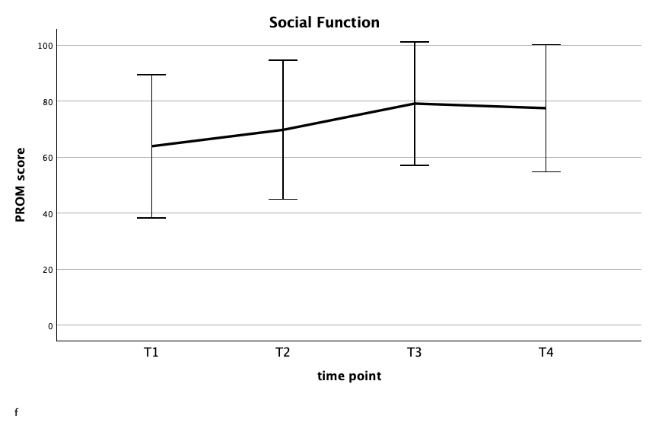

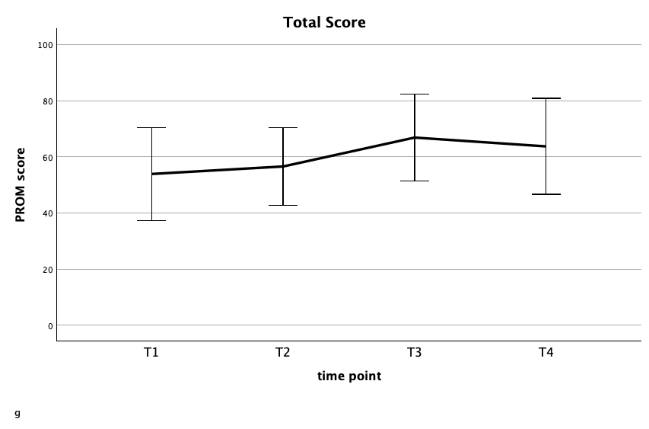


Longitudinal Development of FaCE subscales and Total Score (mean ± SD) Across Four Time Points

Each panel depicts mean PROM scores (0-100) ± SD. Significant improvements from T2 to T3 were observed in all subscales except Oral Function and Eye Comfort. From T3 to T4, a slight decline was noted in Facial Comfort and the FaCE Total Score, whereas other subscales remained stable. Patient number per subscale varied slightly (n = 121-131)

Abbreviations: FaCE = Facial Clinimetric Index; T = time point; T1 = baseline; T2 = therapy initiation; T3 = therapy conclusion; T4 = follow-up; PROM = Patient-Reported-Outcome; SD = standard deviation; n = number of patients


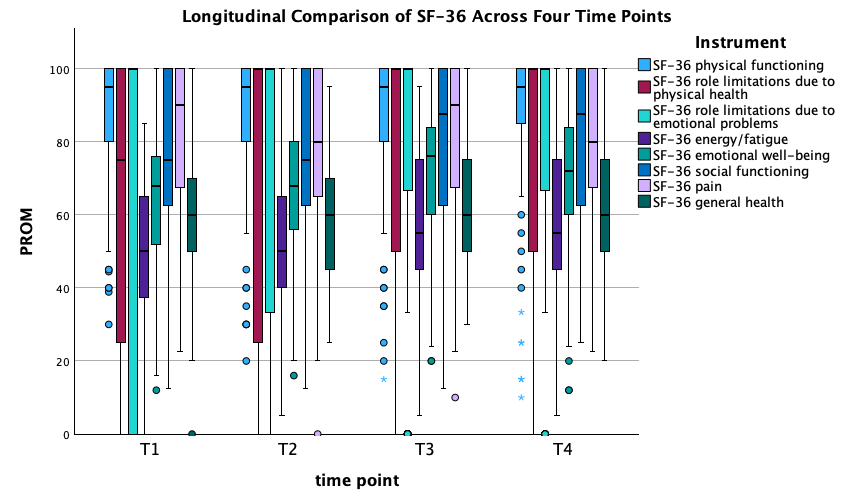


Supplementary Figure 6: SF-36 (n = 127 – 130) across four time points

Longitudinal comparison of SF-36 scores across four time points (T1–T4). Repeated measures ANOVA revealed significant main effects of time for all subscales except Physical Functioning and Pain. Bonferroni-adjusted post-hoc comparisons indicated significant improvements between T2 and T3 in the domains of Energy/Fatigue and Emotional Well-being, suggesting a therapy-related benefit in these aspects of health-related quality of life.

Abbreviations: SF-36 = Short Form-36; PROM = Patient-Reported-Outcome Measure; T = time point; T1 = baseline; T2 = therapy initiation; T3 = therapy conclusion; T4 = follow-up; n = number of patients


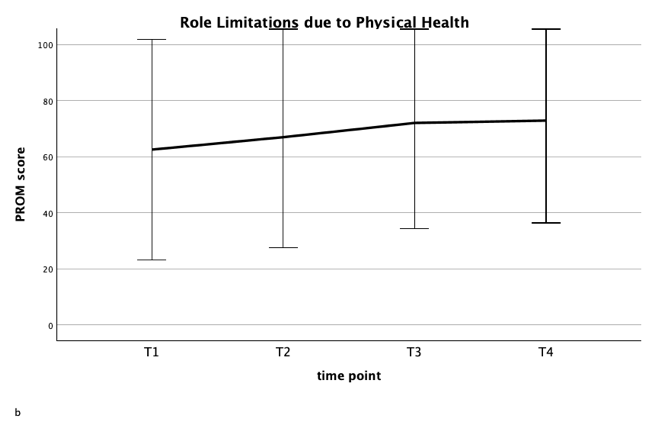

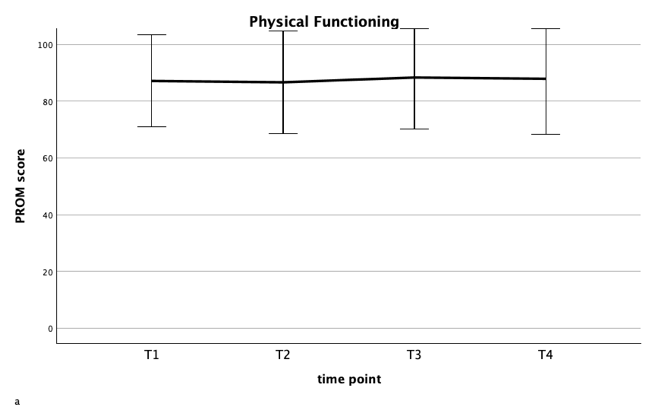

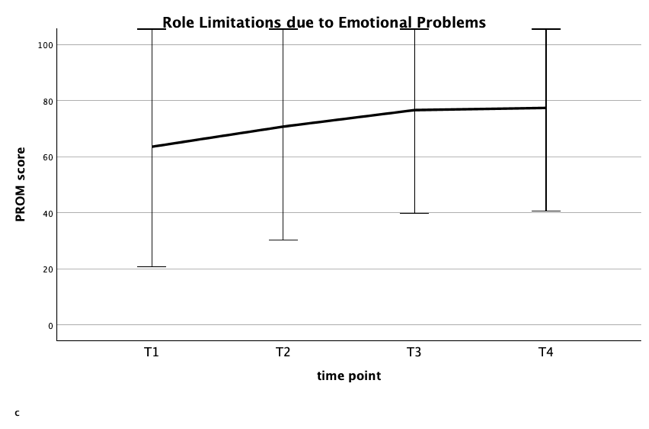

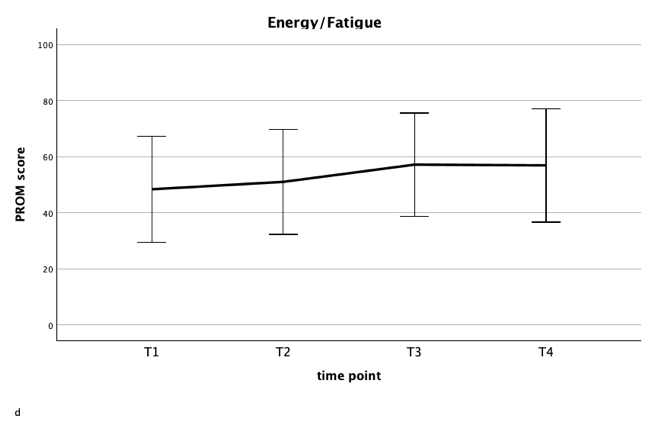

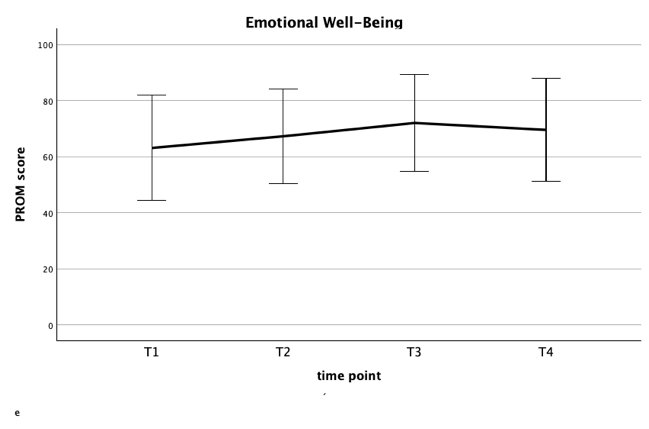

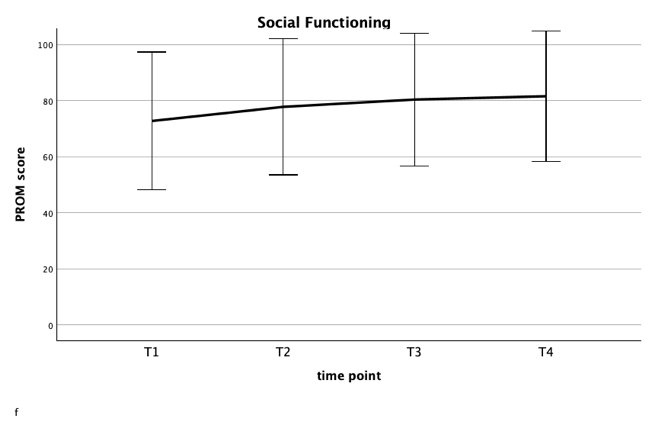

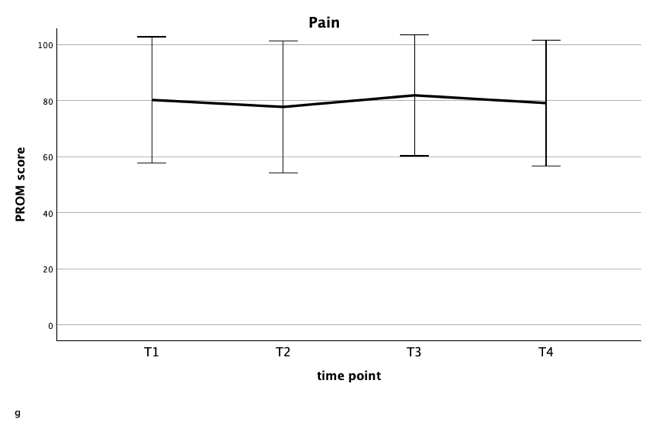

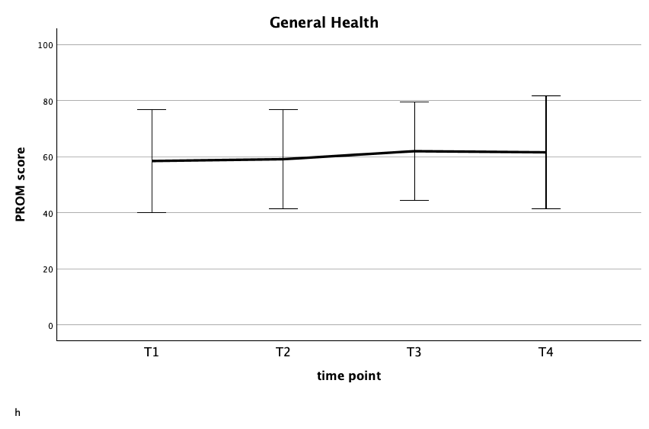


Longitudinal Development of SF-36 Scores (mean ± SD) Across Four Time Points

Supplementary Figure 7: SF-36 Scores across four time points

Significant improvements between T2 and T3 were observed in the subscales Energy/Fatigue, Emotional Well-Being and Pain (alle Bonferroni-adjusted p < 0.05). However, for Pain, the overall repeated measures ANOVA did not reveal a significant main effect of time, suggesting that this finding should be interpreted with caution. No further significant changes were observed between T3 and T4. All other subscales remained stable across time points. Patient number per subscale varied slightly (n = 127-130)

Abbreviations: SF-36 = Short Form-36; T = time point; T1 = baseline; T2 = therapy initiation; T3 = therapy conclusion; T4 = follow-up; PROM = Patient-Reported-Outcome; SD = standard deviation; p = p-Value; ANOVA = analysis of variance; n = number of patients
